# Jaundice and bleeding caused by cystic artery pseudoaneurysm after cholecystectomy: A case report

**DOI:** 10.1097/MD.0000000000043683

**Published:** 2025-08-08

**Authors:** Jiuzheng Sun, Taiyang Zuo, Wenlei Xu, Yadong Wang, Qi Liu, Guanying Yu, Zhao Liu

**Affiliations:** a Department of Hepatobiliary and Pancreatic Surgery, Central Hospital Affiliated to Shandong First Medical University, Jinan, China; b Department of Interventional Oncology, Central Hospital Affiliated to Shandong First Medical University, Jinan, China; c Department of Gastrointestinal Surgery, Central Hospital Affiliated to Shandong First Medical University, Jinan, China.

**Keywords:** biliary hemorrhage, cystic artery pseudoaneurysm, jaundice, laparoscopic cholecystectomy complications, transarterial embolization (TAE)

## Abstract

**Rationale:**

Cystic artery pseudoaneurysm (PSA) after laparoscopic cholecystectomy is a rare but potentially fatal complication that can lead to both jaundice and gastrointestinal bleeding.

**Patient concerns:**

A 42-year-old woman, who was previously asymptomatic and undergoing a routine health examination, presented with jaundice and gastrointestinal bleeding 5 months after her laparoscopic cholecystectomy.

**Diagnoses:**

Initial MRCP raised concerns for bile duct obstruction, prompting consideration of biliary trauma or tumor. However, contrast-enhanced CT and subsequent angiography revealed a 2.5 cm PSA at the previous cystic artery branch, compressing the bile duct.

**Interventions:**

The patient underwent transarterial embolization (TAE) using stainless steel coils to occlude the PSA and control bleeding. This minimally invasive procedure successfully addressed the vascular lesion.

**Outcomes:**

Following TAE, the patient’s jaundice resolved, liver function normalized, and gastrointestinal bleeding ceased. She recovered without complications and remained asymptomatic during a 1-year follow-up.

**Lessons:**

Early multimodal imaging is critical for identifying rare vascular complications, such as cystic artery PSA, following laparoscopic cholecystectomy, while prompt minimally invasive interventions are essential for preventing severe outcomes.

## 1. Introduction

A pseudoaneurysm (PSA) refers to a hematoma formed when the arterial wall is torn or ruptured, allowing blood to escape and become contained by the surrounding tissues, most often due to trauma.^[1]^ Biliary hemorrhage is a relatively rare cause of upper gastrointestinal bleeding, and biliary hemorrhage secondary to the rupture of a cystic artery aneurysm is even rarer.^[2]^ Due to the nonspecific clinical manifestations of cystic artery aneurysms and a limited understanding of the condition, diagnosis is relatively challenging. Rupture of a cystic artery aneurysm may lead to severe complications, including life-threatening massive hemorrhage; thus, timely and accurate diagnosis is crucial.^[3]^ We present a case of jaundice and haemobilia caused by a cystic artery PSA after cholecystectomy. Magnetic resonance cholangiopancreatography (MRCP) initially indicated obstructive jaundice, raising concerns for a portal bile duct tumor or biliary trauma. However, enhanced computed tomography (CT) revealed bile duct compression by a portal hepatic artery aneurysm, which was confirmed by angiography. The patient recovered well following transarterial embolization.

## 2. Case report

A 42-year-old woman presented to the emergency department with complaints of epigastric pain, nausea, jaundice, and multiple episodes of hematemesis, 5 months after undergoing laparoscopic cholecystectomy. The patient reported no history of chronic illnesses or major internal medical conditions, and her postoperative recovery following laparoscopic cholecystectomy had been smooth until the recent onset of symptoms. On physical examination, the patient appeared pale with a blood pressure of 95/52 mm Hg and a heart rate of 102 bpm. Tenderness was noted in the epigastric and right upper quadrant regions. Initial laboratory investigations revealed severe anemia (hemoglobin: 62 g/L), leukocytosis (white blood cell count: 14.87 × 10⁹/L), platelet count was within the normal range (265 × 10⁹/L), and mildly prolonged coagulation parameters. Liver function tests demonstrated elevated total bilirubin (56.9 μmol/L), direct bilirubin (53.3 μmol/L), alanine aminotransferase (89.1 IU/L, reference range: 7–40 IU/L), aspartate aminotransferase (68.5 IU/L, reference range: 13–35 IU/L), γ-glutamyltransferase (107 IU/L, reference range: <50 IU/L), and alkaline phosphatase (178.1 IU/L, reference range: 38–126 IU/L). C-reactive protein levels were normal.

**Figure 1.**
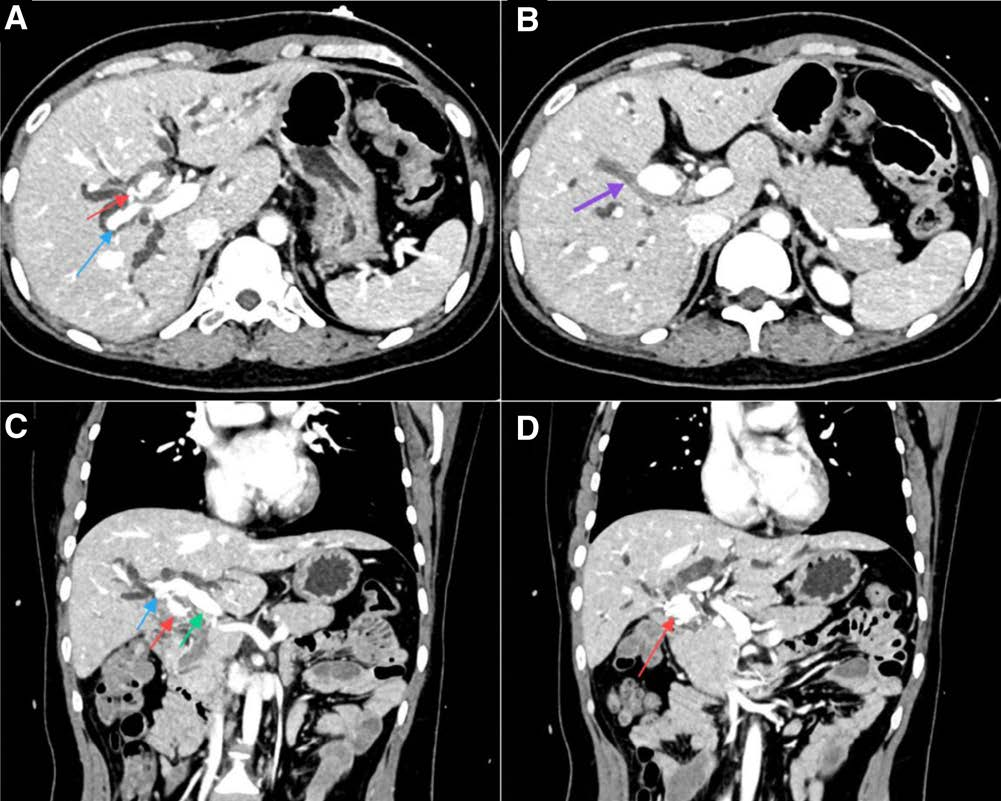
Enhanced CT scan of the chest and abdomen. (A) Axial view showing a high-density lesion (red arrow) at the gallbladder fossa, which is connected to the right hepatic artery (blue arrow). (B) Axial view demonstrating significant dilation of the intrahepatic bile ducts due to compression by the lesion (purple arrow). (C) Coronal view revealing the lesion (red arrow) originating from the right hepatic artery (blue arrow), a branch of the proper hepatic artery (green arrow). (D) The lesion is located at the site of the previously resected gallbladder. CT = computed tomography.

**Figure 2.**
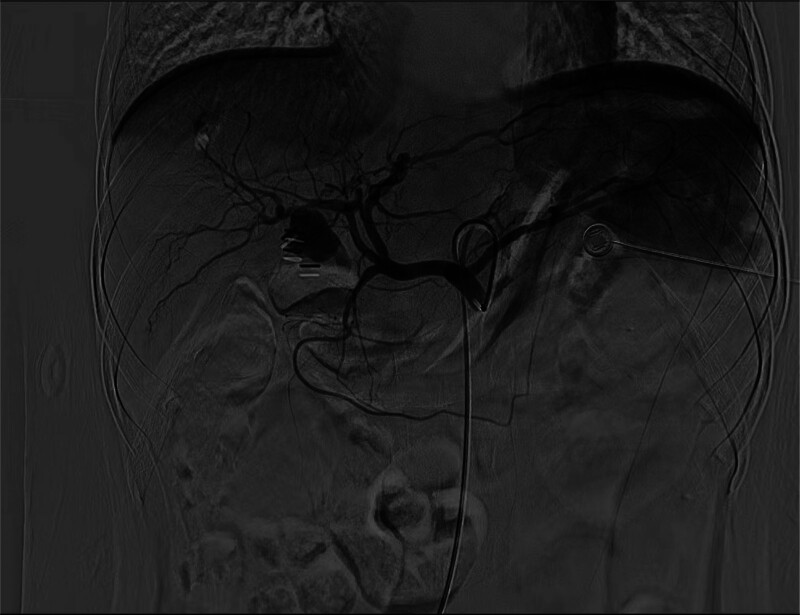
Angiography revealed the celiac trunk branching into the splenic, left gastric, and common hepatic arteries. The right hepatic artery exhibited an irregular vessel wall, with a PSA measuring approximately 2.5 cm at the location of the resected cystic artery branch. PSA = pseudoaneurysm.

MRCP identified bile duct dilation indicative of obstruction at the hepatic hilum. Further imaging with contrast-enhanced CT revealed a 19 × 15 mm hyperdense mass in the gallbladder fossa, closely associated with a clip placed during the previous cholecystectomy and connected to the adjacent right hepatic artery. Additionally, significant dilation of both intrahepatic and common bile ducts was observed (Fig. 1). Subsequent angiography revealed the celiac trunk branching into the splenic artery, left gastric artery, and common hepatic artery. The common hepatic artery was observed to further bifurcate into the gastroduodenal artery, left hepatic artery, and right hepatic artery. The right hepatic artery exhibited an irregular wall structure, with a significant PSA, approximately 2.5 cm in diameter, identified at the previous cystic artery branch site (Fig. 2). This confirmed that the lesion originated from the location of the resected cystic artery.

**Figure 3.**
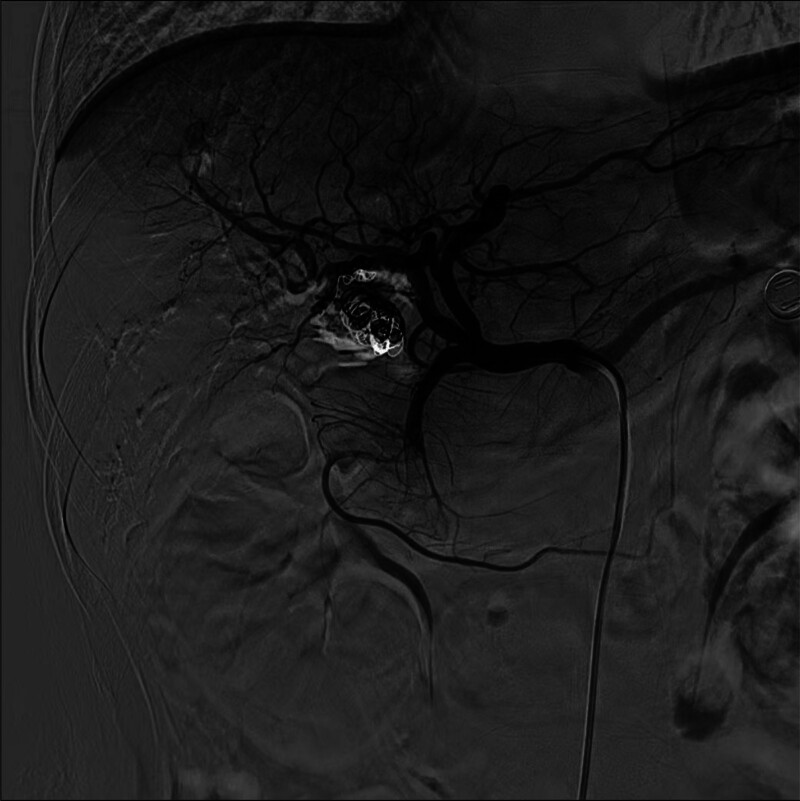
Multiple coils within the PSA effectively arresting blood flow. PSA = pseudoaneurysm.

The patient underwent transarterial embolization using stainless steel coils to successfully occlude both the PSA and the right hepatic artery (Fig. 3). The procedure was successful, and her liver function tests gradually returned to normal. Postoperatively, the patient’s jaundice and gastrointestinal bleeding resolved, and her anemia improved. She experienced no further episodes of hematemesis or melena. Recovery was uneventful, and the patient remained asymptomatic during follow-up evaluations. The procedure was successful. Postoperatively, the patient’s liver function tests gradually returned to normal, her jaundice and gastrointestinal bleeding resolved, and her anemia improved. She experienced no further episodes of hematemesis or melena. Recovery was uneventful, and the patient remained asymptomatic during a 1-year follow-up period.

## 3. Discussion

With the advancement of minimally invasive techniques, laparoscopic cholecystectomy has become an extremely common surgical approach for treating gallbladder stones. Among postoperative complications, the formation and rupture of hepatic or cystic artery PSAs resulting in hemorrhage are exceptionally rare.^[4]^ These PSAs primarily arise due to vascular injuries during surgery, such as clip misplacement, mechanical or thermal trauma, or as a consequence of bile leakage and postoperative infection. These factors weaken the arterial wall, resulting in PSA formation under arterial pressure.^[5]^ Iatrogenic causes account for approximately two-thirds of reported cases, with most PSAs presenting within 8 weeks after the surgical procedure.^[6]^ Notably, our case is exceptional, with the patient developing symptoms 20 weeks postoperatively, which is beyond the typical presentation window.

The clinical manifestations of PSAs lack specificity, and clinicians often misdiagnose or fail to diagnose the condition due to insufficient understanding. Imaging modalities play a pivotal role in the diagnosis of hepatic and cystic artery PSAs. Contrast-enhanced CT is invaluable for identifying vascular abnormalities, such as arterial dilation and PSA formation, while MRCP provides detailed visualization of bile duct obstruction.^[5,6]^ In this case, MRCP revealed bile duct obstruction, suggesting a possible biliary pathology, but the enhanced CT findings confirmed the vascular nature of the lesion. The complementary use of CT and MRCP enabled accurate diagnosis and characterization of the obstruction, highlighting the importance of employing multimodal imaging techniques in similar clinical scenarios. Angiography remains the gold standard for both diagnosing and managing hemobilia. Angiography is the preferred method for identifying the site and cause of bleeding, while also allowing for embolization therapy. Transarterial embolization (TAE) has proven to be a successful and minimally invasive therapeutic option, with reported success rates ranging from 80% to 100%.^[7]^ Surgical intervention is reserved for cases where embolization fails or complications such as infection, arteriovenous fistula, or severe biliary obstruction are present.^[5,8]^

To prevent the formation of hepatic or cystic artery aneurysms, based on over a decade of single-center surgical experience, several key measures are recommended. First, it is crucial to maintain a safe distance from titanium ligation clips during electrocautery to avoid damaging the arterial wall. Additionally, the electric hook should be used cautiously and kept away from the clips to minimize the risk of vascular injury. Furthermore, when intraoperative bleeding occurs from cystic artery branches, blindly employing electrocautery for hemostasis should be avoided. Instead, careful management is advised. Moreover, the use of the electric hook near the hepatoduodenal ligament should be limited to reduce thermal damage in this sensitive area. Finally, surgeons must ensure a thorough understanding of normal hepatic and cystic artery anatomical variations. This includes precise dissection of Calot triangle during surgery to minimize the risk of vascular complications.

## Author contributions

**Conceptualization:** Zhao Liu.

**Funding acquisition:** Zhao Liu.

**Resources:** Wenlei Xu.

**Writing – original** draft: Jiuzheng Sun, Taiyang Zuo.

**Writing – review & editing:** Wenlei Xu, Yadong Wang, Qi Liu, Guanying Yu, Zhao Liu.
